# Mitochondrial and immune response dysregulation in melanoma recurrence

**DOI:** 10.1002/ctm2.1495

**Published:** 2023-11-21

**Authors:** Leticia Szadai, Jéssica de Siqueira Guedes, Nicole Woldmar, Natália Pinto de Almeida, Ágnes Judit Jánosi, Ahmad Rajeh, Ferenc Kovács, András Kriston, Ede Migh, Guihong Wan, Nga Nguyen, Henriett Oskolás, Roger Appelqvist, Fábio CN Nogueira, Gilberto B Domont, Kun‐Hsing Yu, Eugene R. Semenov, Johan Malm, Melinda Rezeli, Elisabet Wieslander, David Fenyö, Lajos Kemény, Peter Horvath, István Balázs Németh, György Marko‐Varga, Jeovanis Gil

**Affiliations:** ^1^ Department of Dermatology and Allergology University of Szeged Szeged Hungary; ^2^ Clinical Protein Science & Imaging, Biomedical Centre Department of Biomedical Engineering Lund University Lund Sweden; ^3^ Chemistry Institute Federal University of Rio de Janeiro Rio de Janeiro Brazil; ^4^ Department of Dermatology Massachusetts General Hospital Harvard Medical School Boston Massachusetts USA; ^5^ Synthetic and Systems Biology Unit Biological Research Centre Eötvös Loránd Research Network Szeged Hungary; ^6^ Department of Biomedical Informatics Harvard Medical School Boston Massachusetts USA; ^7^ Section for Clinical Chemistry Department of Translational Medicine Lund University Lund Sweden; ^8^ Department of Biochemistry and Molecular Pharmacology Institute for Systems Genetics New York University Grossman School of Medicine New York USA; ^9^ Chemical Genomics Global Research Lab Department of Biotechnology College of Life Science and Biotechnology Yonsei University Seoul South Korea; ^10^ First Department of Surgery Tokyo Medical University Tokyo Japan

Dear editor,

Our study delineates the critical influence of the tumour microenvironment on the recurrence of primary melanomas, spotlighting novel biomarkers and potential therapeutic avenues anchored in complex molecular dynamics. This exploration leverages the untapped potential of mitochondrial functions, immune responses and AI‐powered digital pathology to improve early‐stage melanoma prognosis, which accounts for half of melanoma‐specific fatalities worldwide.^1^

Considering the escalating global incidence of melanoma, the current limitations in diagnostic markers necessitate a shift towards more precise predictive models for disease trajectory and personalized melanoma treatment efficacy. Our investigation stands at the forefront of this transformation, employing artificial intelligence and spatial proteomics to study the molecular interplay between tumour cells and their microenvironment in the context of melanoma recurrence. Building upon our foundational work in the Human Melanoma Proteome Atlas, which extensively characterized melanoma presentations and molecular pathology, this study represents a significant advancement.^2^, ^3^, ^4^ We now focus on a more targeted approach, isolating specific tumour regions to intricately investigate the interplay between tumour cells and the stromal component. This progression in our research strategy deepens our molecular understanding of the tumour microenvironment's significance in melanoma recurrence.

We conducted an in‐depth analysis of 12 early‐stage primary melanoma samples (AJCC8 IA‐IIA at diagnosis) procured between 2006 and 2017, aiming to elucidate the molecular and histopathological markers associated with disease recurrence within a 5‐year period postdiagnosis (Figure 1). The subjects were categorized into two groups: one with recurrence (*n* = 6) and another without (*n* = 6). Clinically relevant parameters were documented and summarized in Table S1A and B.

**FIGURE 1:**
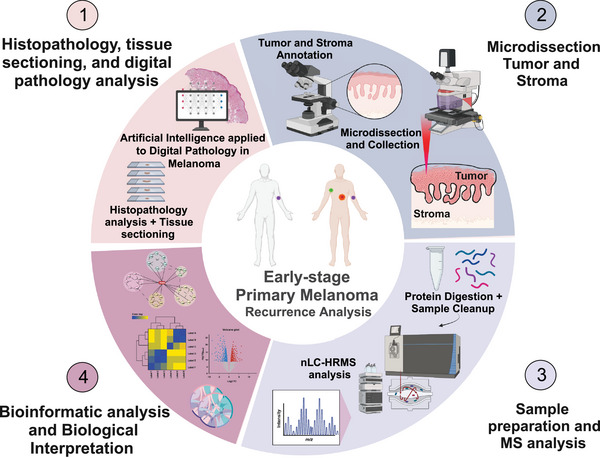
Study concept and workflow. (1) Tissue sectioning and histopathology investigation combined with image analysis based on artificial intelligence (AI). (2) Tumour and stroma annotation followed by laser microdissection. (3) Sample preparation and HR‐DIA‐MS‐based proteomics analysis. (4) Bioinformatic analysis and data biological interpretation.

Our histopathological evaluation demonstrated a clear correlation between tumour thickness (Breslow and Clark indexes) and recurrence, reinforcing their established prognostic significance.^5^ We observed variability in tumour thickness and histopathological characteristics across our cohorts, with the recurrent group showing generally higher Clark levels and Breslow thickness. Additionally, tumours from both groups displayed a range of features characterized by SSM with disparate cellular proliferation, vascular development and immune cell infiltration (Figure S1). Although, these parameters alone did not act as conclusive markers of high recurrence risk. Our analysis of clinical data underscored that Breslow level (Mann–Whitney *U* test: *p* = .0022) and clinical stages (IA–IIA) (Fisher exact test: *p* = .0606) influence recurrence (Table S1). This suggests that although specific histopathological features correlate with recurrence, they are not definitive prognostic markers for early‐stage melanomas, highlighting the need for additional molecular profiling to enhance risk stratification.

We employed AI‐DP using the BIAS v.1.1.1 (Single‐Cell Technologies), which integrates deep Learning and machine learning algorithms, to enhance the accuracy of identifying tumour and stroma cells in early‐stage primary melanomas from histological images.^6^ We trained the algorithmic pipeline to achieve an overall segmentation accuracy of approximately 80% (Recurrence: 81.7 ± 6.3%, no recurrence 78.0 ± 3.6%), corroborated by certified pathologists. Importantly, this AI‐driven methodology was equally effective in discerning normal tissue from stromal and tumour regions across both recurrent and nonrecurrent primary melanomas (Figures 2 and S2).

**FIGURE 2:**
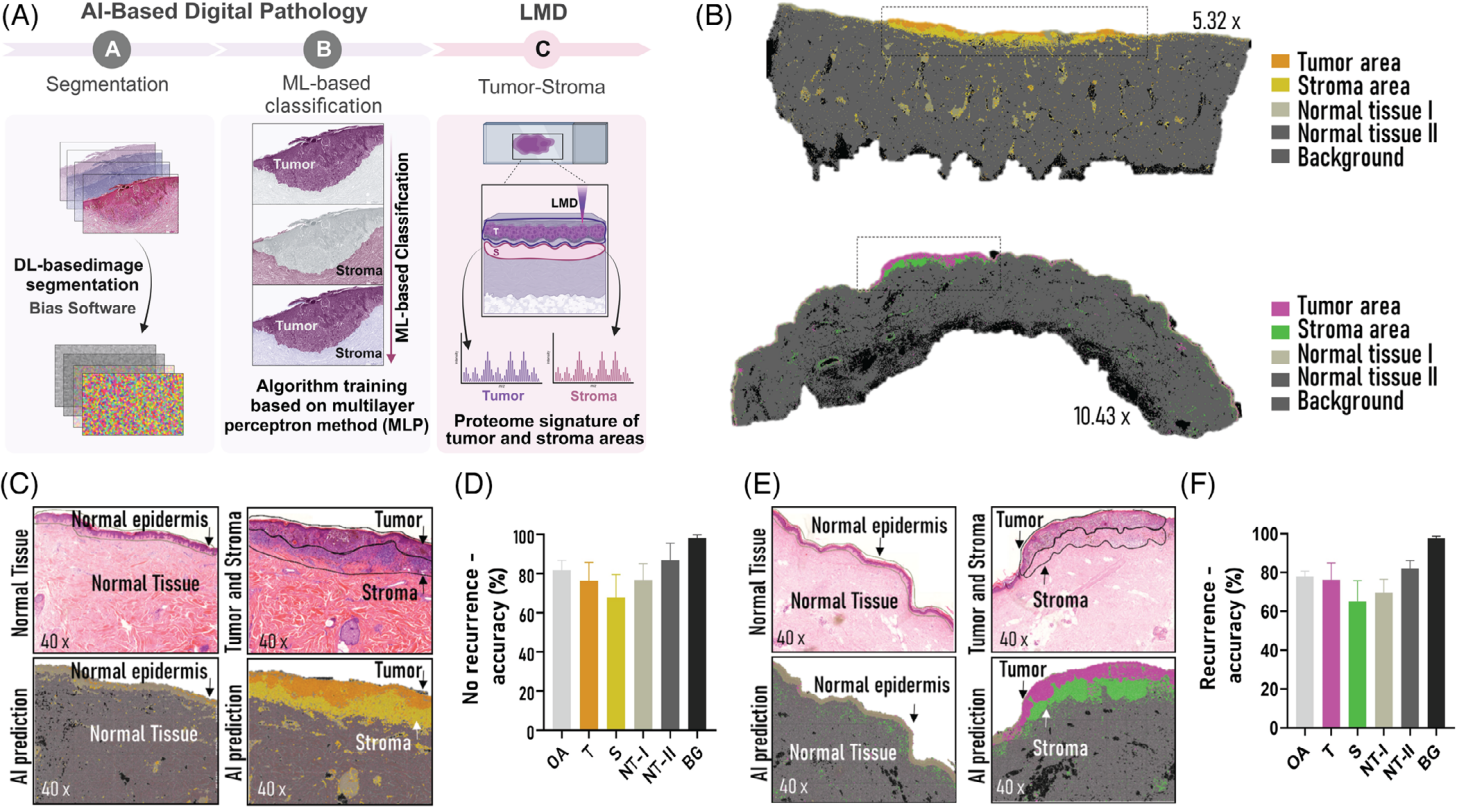
Laser microdissection and digital pathology analysis. (A) Integrative image analysis performed by BIAS software based on deep learning (DL) and machine learning (ML) approaches to identify tumour and stroma areas automatically. Segmentation of FFPE tissue images based on DL analysis. Algorithm training based on ML analysis. Laser capture microdissection and proteome analysis of tumour (T) and stroma (S) regions from primary melanomas with or without regression status. Representative tumour‐stroma prediction based on AI‐DP approach from nonrecurrent and recurrent melanomas. (B) AI prediction of tumour and stroma areas of a nonrecurrent (top) and a recurrent (bottom) primary melanoma tissue. (C) Comparison between manual annotation of normal tissue area and tumour‐stroma area with haematoxylin‐eosin staining and AI‐prediction in nonrecurrent melanoma. (D) AI accuracy (%) prediction for nonrecurrent melanomas. (E) Comparison between manual annotation of normal tissue area and tumour‐stroma area with haematoxylin‐eosin straining and AI‐prediction in recurrent melanoma. (F) AI accuracy (%) prediction for recurrent melanoma sample. Data: OA, overall; T, tumour; S, stroma; NT‐I, normal tissue I (normal epidermis and glands); NT‐II, normal tissue II (dermis and connective tissue); BG, tissue background. Error bars represent relative standard deviation (RSD %) among the samples present in the group.

Subsequently, these specimens were subjected to laser microdissection for the isolation of tumour and stroma components, which were prepared for quantitative proteomics following antigen retrieval and protein digestion.^7^ Utilizing HR‐DIA‐MS, we achieved a comprehensive proteome profiling, confidently identifying over 7000 proteins across all samples (Table S2).

Hierarchical clustering and PLS‐DA analysis revealed distinct proteomic signatures between tumour and stromal compartments, with the stroma exhibiting enhanced differentiation between recurrence status groups (Figure 3A and B). This was further underscored by subsequent ANOVA analyses highlighting the pivotal role of the tumour microenvironment in melanoma recurrence.

**FIGURE 3:**
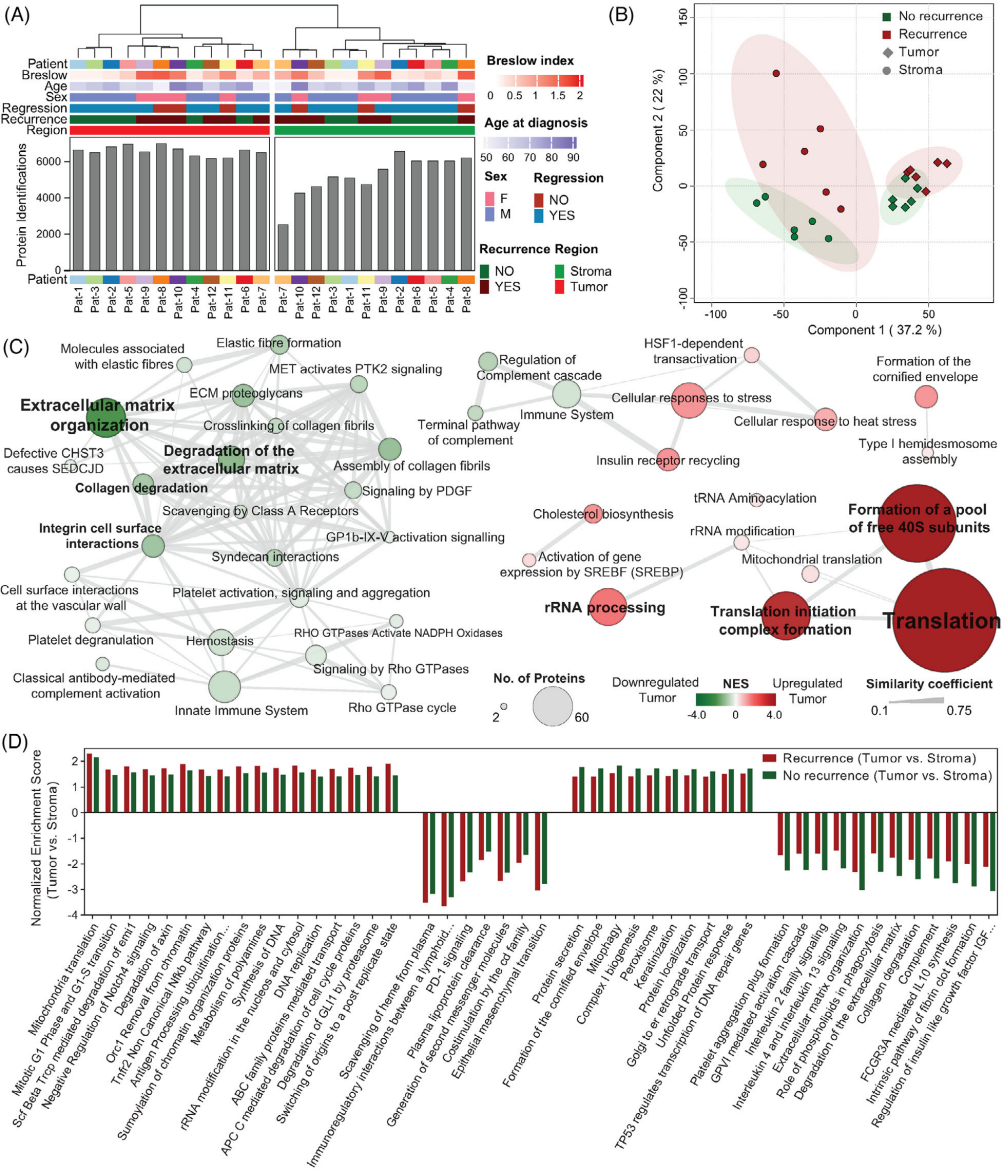
Proteome profiling of tumour cells and stromal regions from recurrent and nonrecurrent primary melanomas. (A) Hierarchical clustering of samples based on their proteome abundance profiles. The bar chart corresponds to the number of proteins identified in each sample. (B) PLS‐DA using the proteomic data on the sample cohort. Proteins included had at least 70% valid values in the entire cohort; the remaining missing data were imputed. (C) Functional annotation enrichment analysis of the fraction of the proteome significantly dysregulated between tumour cells and their microenvironment in both recurrent and nonrecurrent primary melanomas. Annotations in red correspond to upregulated in tumour cells compared to their microenvironment and in green downregulated. (D) GSEA of the proteome differences between tumour cells and stromal regions in both groups of patients. The bar chart represents the normalized enrichment score for the most divergent significantly dysregulated functional annotations between recurrent and nonrecurrent groups. Positive scores indicate upregulated while negative scores indicate downregulated in tumour cells compared to the microenvironment. The analysis on recurrent primary melanomas is represented by red bars while green bars correspond to the analysis on nonrecurrent melanomas.

Functional annotation on the differentially expressed proteome between tumour and stromal regions reveals the specific signatures on a spatial resolution (Figure 3C). Moreover, GSEA highlighted distinct molecular processes in tumour and stromal cells across recurrent and nonrecurrent melanomas. While tumour cells in recurrent cases showed higher enrichment in proliferation‐related pathways like DNA synthesis and mitochondrial translation, the stromal component showed elevated levels of epithelial‐mesenchymal transition and PD‐1 signalling pathways. In contrast, tumour cells from nonrecurrent melanomas exhibited higher enrichment in keratinization and mitophagy pathways, while the stromal region was characterized by features like interleukin signalling and collagen degradation (Figure 3D). These discoveries provide key insights into the distinct molecular mechanisms underlying melanoma recurrence.

GSEA on the proteome differences between tumour cells from recurrent and nonrecurrent melanomas highlighted mitochondrial function as a significant factor in melanoma recurrence. In recurrent cases, tumour cells displayed upregulated mitochondrial pathways and cellular proliferation mechanisms, whereas nonrecurrent cases primarily exhibited immune response pathways (Figures 4A and S3). While our study cohort for nonrecurrent melanoma consisted exclusively of male, it is important to note that the identified risk factors for recurrence, characterized by downregulated immune response pathways, are considered gender‐independent markers of recurrence risk.^4^ Among the 166 significantly dysregulated proteins between tumour cell groups, a cluster of proteins involved in collagen formation, complement and coagulation cascades and immune response activities stood out with the highest score (Figure 4B). Additional immune response markers like CD1A were downregulated in recurrent cases.^8^ Upregulated proteins in recurrent melanomas, such as mitochondrial ADP/ATP translocases ANT1, ANT2 and ANT3, and melanoma cell adhesion molecule (MCAM), suggest a phenotype of increased aggression and higher recurrence risk (Figure 4C). Additionally, the upregulation of the heterogeneous nuclear ribonucleoprotein A1 (HNRNPA1) points to metabolic reprogramming in recurrent tumours,^9^ highlighting the complexity of the interplay between mitochondrial activity, cellular proliferation and immune functions in melanoma recurrence.

**FIGURE 4:**
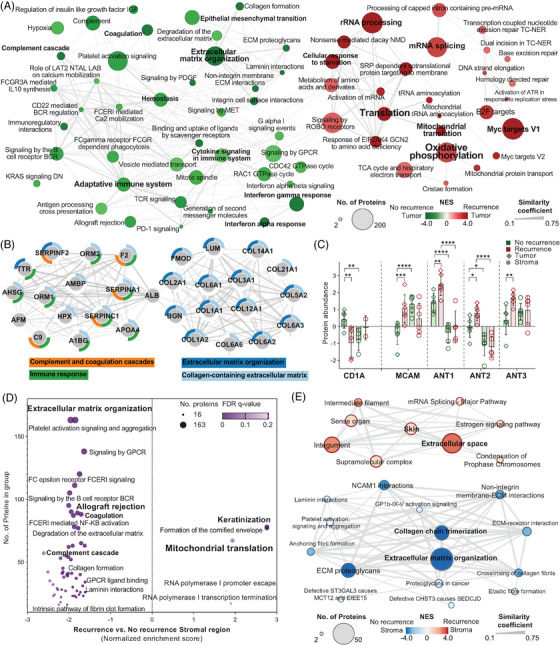
Primary melanoma recurrence is driven by mitochondrial translation and oxidative phosphorylation upregulation and suppression of immune system response. (A) GSEA of the proteome dynamics between tumour cells from recurrent and nonrecurrent primary melanomas. (B) Interaction cluster and functional annotation of proteins significantly downregulated in tumour cells from the recurrence group. (C) Relative abundance of CD1A, MCAM, ANT1, ANT2 and ANT3, bars and error lines correspond to median and standard deviation, respectively. (D) Gene set enrichment analysis of the proteome dynamics in the microenvironment between recurrent and nonrecurrent melanomas. (E) Functional enrichment analysis (STRING) on the significantly dysregulated proteins in the microenvironment between recurrence and no recurrence groups.

Functional pathway analysis revealed that both tumour and stromal regions in recurrent melanomas are enriched in mitochondrial translation (Figure 4), suggesting a shared role in driving recurrence. Notably, the microenvironment in recurrent cases, as compared to nonrecurrent, exhibited significant downregulation of immune response pathways, including extracellular matrix organization and immune cell receptor signalling, mirroring the changes in the tumour region (Figure 4D and E). These results highlight the tumour‐stroma interaction and underscore the importance of the microenvironment in immune evasion and recurrence. Further studies are needed to explore whether these molecular changes are induced or transferred from tumour cells.

Mitochondrial dysfunction, pivotal in melanoma, extends beyond energy metabolism to influence the tumour microenvironment's biological crosstalk. Altered mitochondrial function disrupts metabolic signalling, reactive oxygen species production and apoptotic regulation, potentially leading to immune evasion in recurrent melanomas. Therefore, targeting mitochondrial pathways may offer strategic therapeutic avenues to intercept the metabolic rewiring promoting melanoma recurrence.

As a complementary approach to our proteomic findings, we explored the TCGA data to elucidate the role of mitochondrial pathways at the genomic and transcriptomic levels in early‐stage melanoma.^10^ Multivariate Cox regression identified two mitochondrial pathways − activation and translocation of the BH3‐only proteins NOXA and PUMA − as significantly associated with poorer progression‐free survival (PFS) (Table S3). Although these proteins are traditionally seen as proapoptotic, their association with poorer PFS might reflect an adaptive oncogenic process within the tumour microenvironment. Specifically, the upregulation of transcripts involved in NOXA and PUMA cascades could be an attempt to counteract an environment rich in anti‐apoptotic signals, suggesting a dysregulated apoptotic signalling pathway. However, our proteomic data did not exhibit a corresponding upregulation at the protein level, indicating a potential posttranscriptional regulatory mechanism that could impede the translation of these proapoptotic signals into functional proteins. This divergence between transcriptomic and proteomic findings emphasizes the intricacies of melanoma biology and the importance of integrating multiomics data to gain a comprehensive understanding of the disease's molecular landscape.

In conclusion, our investigation highlights the critical influence of the tumour microenvironment in melanoma recurrence, advancing the current scope of early‐stage melanoma research. By integrating spatial proteomics with AI‐driven digital pathology, we identified novel risk markers pertinent to melanoma recurrence. These findings contribute to the risk stratification for disease recurrence and suggest targeted therapeutic strategies focusing on mitochondrial functionality and immune checkpoints, expanding personalized melanoma treatment.

## AUTHOR CONTRIBUTIONS

Study concept: L.S., J.S.G., I.B.N., G.M.‐V., and J.G.; Methodology: L.S., J.S.G., N.W., N.P.A., A.J.J., A.R., G.W., M.R., and J.G.; Software and images visualization: J.S.G., F.K., A.K., E.M. and P.H.; Data analysis: L.S., J.S.G., N.W., and J.G.; Collected patients. samples: L.S., H.O., and I.B.N.; Supervision: R.A., F.CN.N., G.B.D., K.‐H.Y., E.R.S., J.M., E.W., D.F., L.K., P.H., I.B.N., G‐M.‐V., and J.G.; Writing.original draft: L.S., J.S.G., A.J.J., and J.G.; Writing.final version: J.S.G., G.M.‐V., and J.G. Review and editing: all authors; Scientific oversight of the study: all authors.

## CONFLICT OF INTEREST STATEMENT

The authors declare no potential conflicts of interest.

## ETHICAL APPROVAL

The study workflow was approved by the Hungarian Ministry of Human Resources, Department of Health Administration, and the Deputy State Secretary for the National Chief Medical Officer (Approval number: 4463‐6/2018/EÜIG) and conducted following relevant guidelines and regulations from the Swedish biobanking laws and Declarations of Helsinki.

## Supporting information

Supporting Information

Supporting Information

Supporting Information

Supporting Information

Supporting Information

Supporting Information
