# Supplementary material for: Mitochondrial and immune response dysregulation in melanoma recurrence
Source: Clin Transl Med. 2023 Nov 21;13(11):e1495. doi: 10.1002/ctm2.1495 (PMC10663649; doi:10.1002/ctm2.1495)
Supplement: Supplementary file 1 — Supporting Information [file CTM2-13-e1495-s001.docx]

**Supplemental figures:**


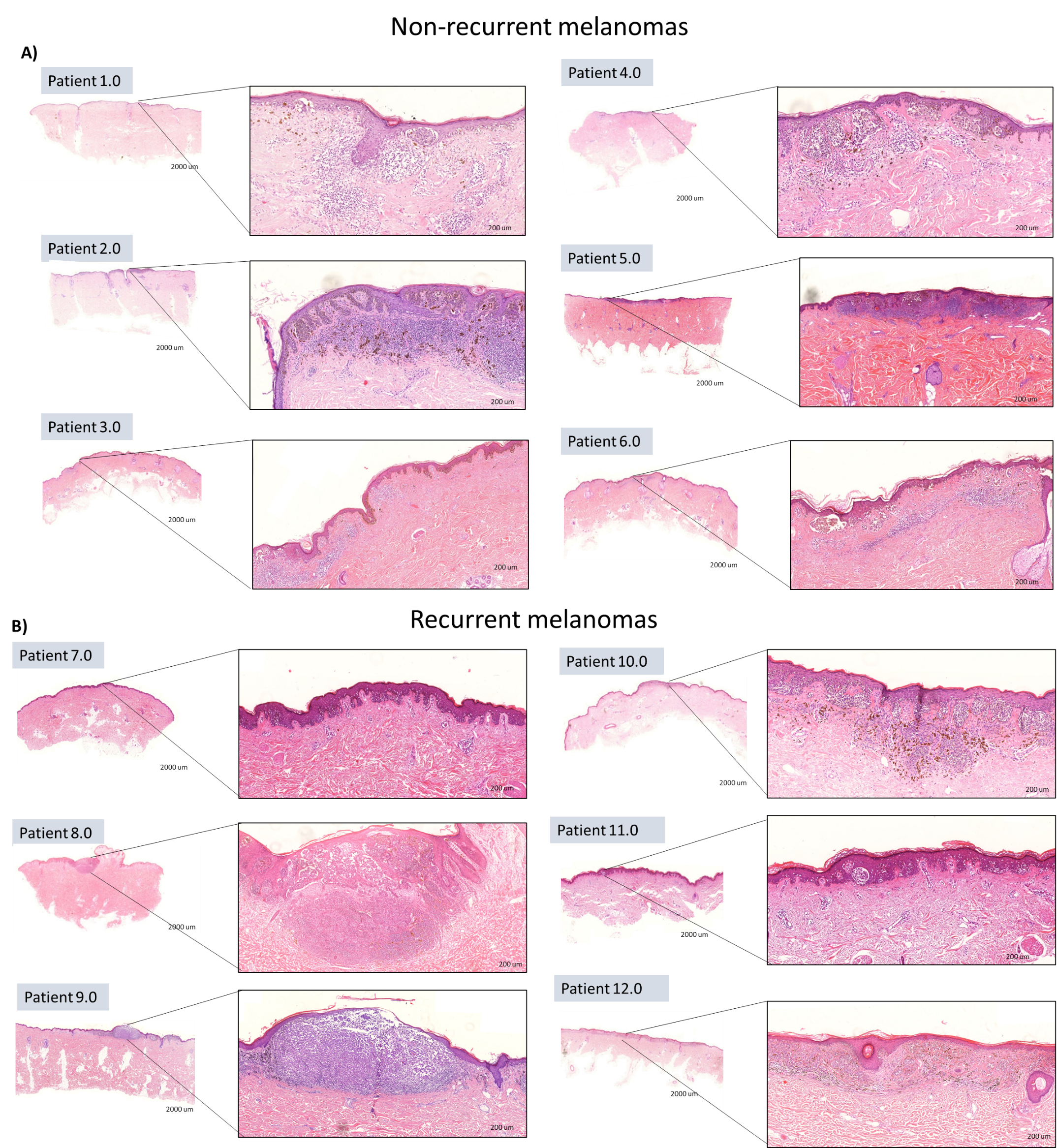


**Figure S1. Representative hematoxylin-eosin (H&E)-stained slides of tumor tissue from the 12 primary melanomas included in the study.** A) corresponds to non-recurrent melanomas, and B) corresponds to recurrent melanomas. The magnified slides show the tumoral and stromal parts in the layers of the epidermis and dermis. Images on the left side of the figure are at a magnification of 2000 µm, while those on the right side are at a magnification of 200 µm.


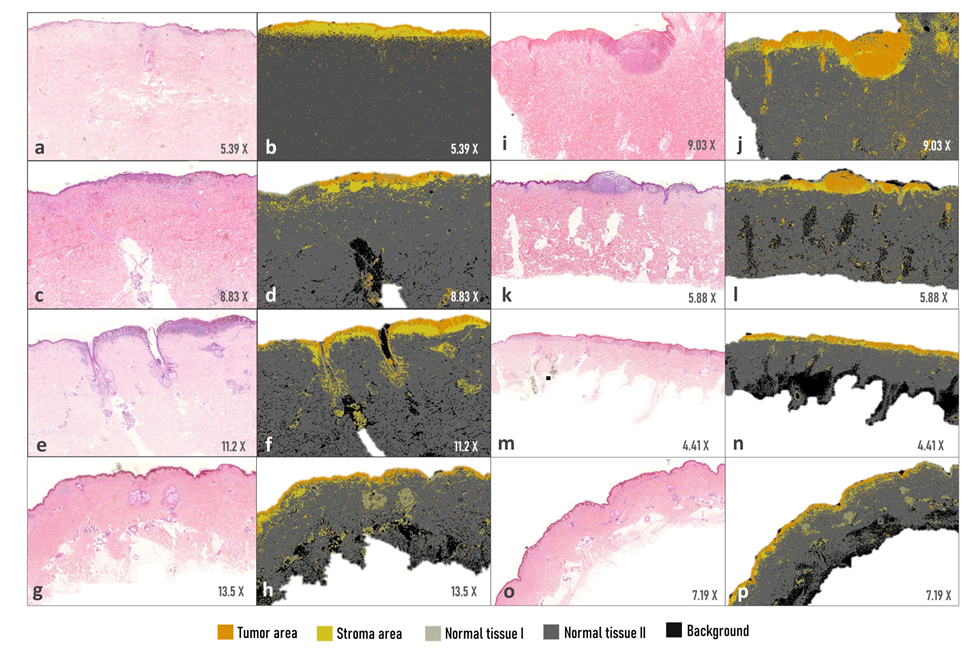


**Figure S2. Representative tumor-stroma prediction based on AI-DP approach from non-recurrent and recurrent melanomas.** A, C, E, G, and O) H&E-stained scanning images used to train algorithms to automatically identify and distinguish tumor and stroma areas in non-recurrent early-stage primary melanomas. B, D, F, H, and P) AI-prediction of tumor (Dark yellow) and stroma (Light yellow) areas of the correspondent non-recurrent primary melanoma tissues. I, K, and M) H&E-stained scanning images used to train algorithms to automatically identify and distinguish tumor and stroma areas in recurrent early-stage primary melanomas. J, L, and N) AI-prediction of tumor (Dark yellow) and stroma (Light yellow) areas of the correspondent recurrent primary melanoma tissues. The overall accuracy prediction was about 80%. Data: Normal tissue I (normal epidermis and glands), Normal tissue II (dermis and connective tissue).


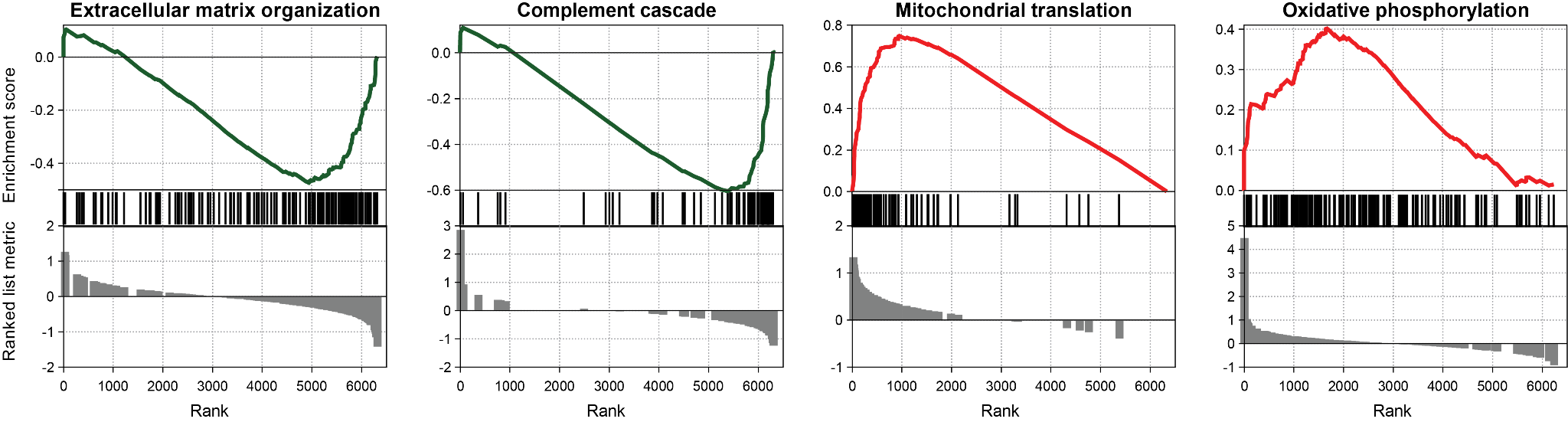


**Figure S3. Gene Set Enrichment Analysis (GSEA) plots of pathways significantly dysregulated between tumor cells from patients grouped based on their recurrence status.**
