# Supplementary material for: Mitochondrial and immune response dysregulation in melanoma recurrence
Source: Clin Transl Med. 2023 Nov 21;13(11):e1495. doi: 10.1002/ctm2.1495 (PMC10663649; doi:10.1002/ctm2.1495)
Supplement: Supplementary file 2 — Supporting Information [file CTM2-13-e1495-s002.docx]

**Supplemental information**

**Methods**

## Cohort description and ethical considerations

Twelve patients with melanoma of the skin were enrolled in the study after presenting with their primary lesion to the Department of Dermatology and Immunology at the University of Szeged between 2006 and 2017. Samples of the primary lesions were obtained after surgical excision and stored as FFPE tumor blocks. Clinicopathological data, including age, gender, ulceration, Breslow and Clark levels, clinical stage, histopathological type, presence of tumor regression, and survival parameters (DFS, PFS, and OS), were summarized in **Table 1** and supplementary material (**Table S1**).

DFS, PFS, and OS were calculated for the patient group with melanoma recurrence based on the date of clinical diagnosis of the primary melanoma to the date of first metastasis, progression, and death or last follow-up, respectively. For the group without recurrence, survival parameters were calculated from the date of primary melanoma diagnosis to the date of the last follow-up.

The primary tumor samples were divided into two groups: one from patients who experienced disease recurrence within 5 years after the initial diagnosis (n=6), and the other from those who had no metastatic events within that same 5-year period (n=6). All of these tumor samples were from the early stages of the disease, specifically classified as AJCC8 IA-IIA at the time of diagnosis. Non-parametric statistical analysis (Mann-Whitney U test) was performed using GraphPrism software (version 9.5) to compare clinical pathology. A P-value ≤ 0.05 was considered significant.

The study workflow was approved by the Hungarian Ministry of Human Resources, Department of Health Administration, and the Deputy State Secretary for the National Chief Medical Officer (Approval number: 4463-6/2018/EÜIG) and conducted following relevant guidelines and regulations from the Swedish biobanking laws and Declarations of Helsinki.

## Histopathological assessment of the sample cohort

The examined paraffin-archived tumor samples encompassed a range of thickness from thin, low risk (< 1 mm, pT1a) – characteristic of the no recurrence subgroup, to medium thick, medium risk (1–1.6 mm, pT1b-pT2b) – characteristic of the recurrence subgroup. Based on histology subtypes, all tumors exhibited histological features of superficial spreading melanoma (SSM), characterized by differentiated solid tumor nests reaching Clark level III in most samples from the recurrence group (**Table 1**). The analysis unveiled that regression of up to 75% was a common feature in the no-recurrence group, exhibiting proliferation of small vessels, fibrosis, and invasion of lymphoid cells and specialized macrophages; melanophages [1]. Conversely, in the recurrence group, 50% of the tumors displayed similar regression patterns accompanied by outlined lymphocyte-rich peritumoral niches. In the subgroup with no recurrence, two cases exhibited pagetoid spreading of melanoma cells, accompanied by the presence of epithelioid-type melanoma cells. In the subgroup of recurrence, variable pigment production was observed in the epidermis. Moreover, nevoid and epithelioid cells were mixed, and vertical growth of superficial spreading melanoma was observed in one case (Supplementary Information, **Figure S1**). In summary, we observed no significant histopathological differences between recurrent and non-recurrent early-stage melanomas that might indicate a high risk for disease recurrence.

## Histopathological assessment of the sample cohort

The examined paraffin-archived tumor samples encompassed a range of thickness from thin, low risk (< 1 mm, pT1a) – characteristic of the no recurrence subgroup, to medium thick, medium risk (1–1.6 mm, pT1b-pT2b) – characteristic of the recurrence subgroup. Based on histology subtypes, all tumors exhibited histological features of superficial spreading melanoma (SSM), characterized by differentiated solid tumor nests reaching Clark level III in most samples from the recurrence group (Table 1). The analysis unveiled that regression of up to 75% was a common feature in the no-recurrence group, exhibiting proliferation of small vessels, fibrosis, and invasion of lymphoid cells and specialized macrophages; melanophages [1]. Conversely, in the recurrence group, 50% of the tumors displayed similar regression patterns accompanied by outlined lymphocyte-rich peritumoral niches. In the subgroup with no recurrence, two cases exhibited pagetoid spreading of melanoma cells, accompanied by the presence of epithelioid-type melanoma cells. In the subgroup of recurrence, variable pigment production was observed in the epidermis. Moreover, nevoid and epithelioid cells were mixed, and vertical growth of superficial spreading melanoma was observed in one case (Supplementary Information, Figure S1). In summary, we observed noteworthy differences in Breslow (Mann-Whitney U test, p=0.0022) and Clark level (Mann-Whitney U test, p=0.0152) between the two subgroups. However, it's important to highlight that other significant histopathological characteristics differing between recurrent and non-recurrent early-stage melanomas indicating high risk for disease recurrence were not observed.

## FFPE sample preparation and histology analysis

The cohort of primary melanomas (n=12) stored as FFPE tumor blocks underwent a sectioning and staining procedure. The slice thickness was adjusted to 6 µm using a conventional microtome for sectioning. Hematoxylin and eosin (H&E) were used to stain all FFPE samples, which were then scanned automatically. Histological analyses were conducted to classify tumor subtypes and determine general histopathology data such as Breslow and Clark level (see **Table 1**), as well as the tumor-stroma ratio.

## Digital pathology: Annotation method and accuracy assessment

Deep-Learning (DL) and Machine Learning (ML) algorithms were trained on H&E-stained whole FFPE tissue scans for automatic annotation and differentiation of tumor and stromal content in investigated primary Melanomas. Biological Image Analysis software [2] (BIAS, v. 1.1.1, Single-Cell Technologies) was used for integrative image analysis, which integrated image pre-processing, deep-learning-based image segmentation, feature extraction, and machine-learning approaches for tissue part categorization. The superpixels method with the SLICO algorithm was used for segmentation. The features were extracted from the segmented cells with a region size of 40 pixels, ellipse orientation, Haralick texture, and intensity adjustment. The multilayer perceptron method (MLP), a supervised learning method with a feedforward artificial neural network (ANN), was applied in the ML-based classification. The ANN is made up of several layers of perceptrons and at least three node layers: an input layer, a hidden layer, and an output layer. Except for the input nodes, each node is a neuron with a nonlinear activation function. The Sigmoid function is used as the activation function in this method. For training, MLP employed a supervised learning technique known as backpropagation. K-fold cross-validation was used to determine the accuracy level related to the classification of tumor and stroma parts, normal tissue I *(i.e.,* epidermis and glands), and normal tissue II (*i.e.,* connective tissue and dermis) [2, 3].

## Laser-microdissection: tumor and stroma isolation

Previously annotated H&E-stained tissue sections from the 12 patient samples were prepared and transferred for automated laser capture microdissection. This process was conducted to isolate and collect tumor cells and associated stromal regions. The laser microdissection system utilized consisted of a Zeiss PALM MicroBeam, equipped with a laser catapult (Zeiss, Germany). High cutting precision was achieved using wide-field optics with a 10x objective lens, allowing for a laser cut energy range of 70-74. The isolated material was collected through the implementation of a laser pulse into a reaction tube. Annotation and control over the collection of contours were performed using PALMRobo version 4.6 software. For subsequent proteome analysis, approximately 10,000 cells were collected per sample (i.e., tumor: 11083 ± 5241 cells, stroma: 10152 ± 5508 cells), with consideration given to the total area collected and the thickness of the slide.

## Sample preparation for proteomics

The microdissected tissue samples were processed as previously described [4, 5]. Briefly, the isolated regions were incubated with 20 µl of protein extraction buffer (50 mM TEAB, 25 mM DTT, 5% SDS) for 60 minutes at 99°C to induce antigen retrieval. Samples were sonicated using a Bioruptor Plus UCD-300 (Diagenode) for 20 cycles (15 seconds on and 15 seconds off) at 4°C for DNA shearing. Protein digestion was performed using the S-TRAP method with 1 µg of trypsin, as previously reported [6]. The peptide content was determined using a nanodrop (DeNovix DS-11, USA) and immediately submitted to MS analysis. A total of 1 μg of peptides was injected onto the LC-MS system.

## Mass spectrometry-based analysis

The LC-MS system used for analysis consisted of an Ultimate 3000 RSLCnano UPLC (Dionex) coupled to a Q-Exactive HF-X mass spectrometer (Thermo Fisher Scientific, Waltham, MA, USA). The peptide mixture (approximately 1 µg) from each sample was spiked with iRT (Biognosys AG, Schlieren, Switzerland) before analysis. The LC system included a trap column Acclaim PepMap C18 (3 µm, 100 Å, 75 µm i.d. × 2 cm, nanoViper) and an analytical column EASY-spray RSLC C18 (2 µm, 100 Å, 75 µm i.d. × 50 cm). The loading buffer, running at a flow rate of 5 μL/min, was an aqueous solution of 0.1% TFA. The separation buffers were obtained from Thermo Fisher Scientific (Waltham, MA, USA). The temperatures of the trap and analytical columns were set at 35 °C and 60 °C, respectively. The peptide separation was carried out using a non-linear gradient of 100 min. The gradient went from 4% to 26% of B over 85 min, followed by an increase to 50% over 15 min. A variable window data-independent acquisition (DIA) method, recently implemented, was used to acquire the mass spectrometry (MS) data [7].

## Data analysis

The DIA-NN software was used to perform a protein database search on the DIA runs in direct mode. The human reference database from the UniProt repository in 2022 was used. Protein identification and quantification were performed using a label-free approach with an FDR of 1% at both the peptide and protein levels. A total of 7484 proteins were confidently identified in this study. The data was processed using the Perseus platform [8]. The abundance values were log2 transformed and then subtracted by the median of all identified proteins in the sample (**Table S2**). For PLS-DA analysis, 5401 proteins, corresponding to 70% of valid values in the entire cohort, were used, and imputation was performed on the remaining missing data.

To determine significantly dysregulated proteins between groups, statistical analysis was performed using GraphPad Prism 9. Proteins were considered significantly dysregulated if the FDR was 5%, using the Two-stage step-up (Benjamini, Krieger, and Yekutieli) method incorporated in the software. Gene Set Enrichment Analysis (GSEA) was conducted on the entire dataset after normalization and standardization, with HALLMARK and REACTOME Version 2022 gene set databases utilized in the analysis. The results were visualized in Cytoscape V3.9.1. Significantly dysregulated proteins were functionally analyzed using the STRING app in Cytoscape.

## TCGA pathway analysis

We utilized public data from The Cancer Genome Atlas (TCGA) to further validate our main proteomic findings. Survival data for patients with melanoma stage ≤ IIC were obtained from the clinical data resource (TCGA-CDR) [9]. Validated progression-free survival (PFS) was used, which is defined as the period from diagnosis until the first locoregional recurrence, distant metastasis, new primary tumor, or death with tumor. Alive patients without these events were censored [9, 10]. To focus on protein change in the setting of multiple regulatory pathways, we used pathways previously identified by the PARADIGM algorithm [11, 12]. The methodology combines gene expression, copy-number alteration, and interaction data from several databases to identify “SuperPathways.” Each patient had a Z-transformed single-sample gene-set enrichment (ssGSEA) score. Patients without PFS or pathway data were filtered. We only included mitochondrial-related pathways (13 total) based on literature and pathway name. The association of each mitochondrial pathway with PFS was determined using Cox proportional hazard models while including age, sex, and AJCC stage as covariates. Benjamini-Hochberg was used for multiple testing correction.

## Data and code availability

Proteomic data generated in the study were deposited in PRIDE consortium under the accession code: **Will be submitted during revision.**

**References**

1. Guitera P, Li LXL, Scolyer RA, Menzies SW (2010) Morphologic features of melanophages under in vivo reflectance confocal microscopy. Arch Dermatol 146:492–498. https://doi.org/10.1001/archdermatol.2009.388

2. Mund A, Coscia F, Kriston A, et al (2022) Deep Visual Proteomics defines single-cell identity and heterogeneity. Nat Biotechnol 40:1231–1240. https://doi.org/10.1038/S41587-022-01302-5

3. Hollandi R, Szkalisity A, Toth T, et al (2020) nucleAIzer: A Parameter-free Deep Learning Framework for Nucleus Segmentation Using Image Style Transfer. Cell Syst 10:453-458.e6. https://doi.org/10.1016/j.cels.2020.04.003

4. Velasquez E, Szadai L, Zhou Q, et al (2021) A biobanking turning‐point in the use of formalin‐fixed, paraffin tumor blocks to unveil kinase signaling in melanoma. Clin Transl Med 11:e466. https://doi.org/10.1002/ctm2.466

5. Pirhonen J, Szkalisity Á, Hagström J, et al (2022) Lipid Metabolic Reprogramming Extends beyond Histologic Tumor Demarcations in Operable Human Pancreatic Cancer. Cancer Res 82:3932–3949. https://doi.org/10.1158/0008-5472.CAN-22-0396

6. Kuras M, Woldmar N, Kim Y, et al (2021) Proteomic Workflows for High-Quality Quantitative Proteome and Post-Translational Modification Analysis of Clinically Relevant Samples from Formalin-Fixed Paraffin-Embedded Archives. J Proteome Res 20:1027–1039. https://doi.org/10.1021/acs.jproteome.0c00850

7. Gil J, Rezeli M, Lutz EG, et al (2021) An Observational Study on the Molecular Profiling of Primary Melanomas Reveals a Progression Dependence on Mitochondrial Activation. Cancers (Basel) 13:. https://doi.org/10.3390/CANCERS13236066

8. Tyanova S, Cox J (2018) Perseus: A bioinformatics platform for integrative analysis of proteomics data in cancer research. In: Methods in Molecular Biology

9. Liu J, Lichtenberg T, Hoadley KA, et al (2018) An Integrated TCGA Pan-Cancer Clinical Data Resource to Drive High-Quality Survival Outcome Analytics. Cell 173:400-416.e11. https://doi.org/10.1016/J.CELL.2018.02.052

10. Hudis CA, Barlow WE, Costantino JP, et al (2007) Proposal for standardized definitions for efficacy end points in adjuvant breast cancer trials: The STEEP system. J Clin Oncol 25:2127–2132. https://doi.org/10.1200/JCO.2006.10.3523

11. Vaske CJ, Benz SC, Sanborn JZ, et al (2010) Inference of patient-specific pathway activities from multi-dimensional cancer genomics data using PARADIGM. Bioinformatics 26:. https://doi.org/10.1093/bioinformatics/btq182

12. Hoadley KA, Yau C, Hinoue T, et al (2018) Cell-of-Origin Patterns Dominate the Molecular Classification of 10,000 Tumors from 33 Types of Cancer. Cell. https://doi.org/10.1016/j.cell.2018.03.022
